# Supplementary material for: Perceived efficacy of COVID-19 restrictions, reactions and their impact on mental health during the early phase of the outbreak in six countries
Source: R Soc Open Sci. 2020 Aug 12;7(8):200644. doi: 10.1098/rsos.200644 (PMC7481706; doi:10.1098/rsos.200644)
Supplement: Supplementary analyses [file rsos200644supp1.docx]

Perceived efficacy of COVID-19 restrictions, reactions and their impact on mental health during the early phase of the outbreak in six countries

Mækelæ, Reggev et al. 2020, Supplementary material

We here provide additional figures and analyses (post-hoc tests).

### Protective actions for oneself vs for others

Figure S1: The majority of respondents engaged in protective actions like hand washing and social distancing for themselves and others. Some who did engage in hand washing and social distancing might not see it as protective behavior, hence the few who did state doing the action but stated also not performing any protective action.

|  | | | | | | **Hand washing** | | | |  |  |
| --- | --- | --- | --- | --- | --- | --- | --- | --- | --- | --- | --- |
| **Protective Actions Self** | | | | | | **no** | | **yes** | | **Total** |  |
| No protective action self | | | | |  | 25 |  | 210 |  | 235 |  |
| Protective action self | | | | |  | 31 |  | 1994 |  | 2025 |  |
| Total | | | | |  | 56 |  | 2204 |  | 2260 |  |
| Χ² |  | 72.276 |  | 1 |  |  |  |  |  |  |  |

|  | | | | | | | | | | | **Social distancing** | | | | | | | | | |  | |
| --- | --- | --- | --- | --- | --- | --- | --- | --- | --- | --- | --- | --- | --- | --- | --- | --- | --- | --- | --- | --- | --- | --- |
| **Protective Actions Self** | | | | | | | | | | | **no** | | | | **yes** | | | | | | **Total** | |
| No protective action self | | | | | | | | | |  | 102 | |  | | 133 | | | |  | | 235 |  |
| Protective action self | | | | | | | | | |  | 138 | |  | | 1887 | | | |  | | 2025 |  |
| Total | | | | | | | | | |  | 240 | |  | | 2020 | | | |  | | 2260 |  |
| Χ² | |  | | 296.996 | | |  | 1 |  | < .001 |  | | | | |  |  |  |  |  |  |  |
|  | | | | | | | | | | | | | | | | | | | | |  |  |
|  | | | | | | | | | | | | **Hand washing** | | | | | | |  | |  |  |
| **Protective Actions for Others** | | | | | | | | | | | | **no** | | | **yes** | | | | **Total** | |  |  |
| No protective actions for others | | | | | | | | | | |  | 19 |  | | 182 | | |  | 201 |  |  |  |
| protective actions for others | | | | | | | | | | |  | 32 |  | | 1947 | | |  | 1979 |  |  |  |
| Total | | | | | | | | | | |  | 51 |  | | 2129 | | |  | 2180 |  |  |  |
| Χ² | |  | 49.036 |  | 1 | |  | < .001 | | |  |  |  |  |  |  |  |  |  |  |  |  |
|  | | | | | | | | | | | | | | | | | |  |  |  |  |  |
|  | | | | | | | | | | **Social distancing** | | | |  | | | |  |  |  |  |  |
| **Protective Actions for Others** | | | | | | | | | | **no** | | **yes** | | **Total** | | | |  |  |  |  |  |
| No protective actions for others | | | | | | | | |  | 82 |  | 119 |  | 201 | | |  |  |  |  |  |  |
| protective actions for others | | | | | | | | |  | 146 |  | 1833 |  | 1979 | | |  |  |  |  |  |  |
| Total | | | | | | | | |  | 228 |  | 1952 |  | 2180 | | |  |  |  |  |  |  |
| Χ² |  | 217.600 | |  | 1 |  | < .001 |  |  |  |  |  |  |  |  |  |  |  |  |  |  |  |

### Follow-up analysis for Table 3 in the main document

Comparison of psychological variables across countries (GLM and post-hoc tests)

| ANOVA – Perceived Risk per Country | | | | | | | | | | | | | | | | |
| --- | --- | --- | --- | --- | --- | --- | --- | --- | --- | --- | --- | --- | --- | --- | --- | --- |
| **Cases** | | **Sum of Squares** | | | **df** | | **Mean Square** | | | | **F** | | **p** | | **η²** | |
| Countries |  | 26404.248 | |  | 7.000 |  | 3772.035 | | |  | 8.705 |  | < .001 |  | 0.026 |  |
| Residual |  | 970184.370 | |  | 2239.000 |  | 433.311 | | |  |  |  |  |  |  |  |
| Note.  Type III Sum of Squares | | | | | | | | | | | | | | | | |
| **Post Hoc Comparisons - Countries** | | | | | | | | | | | | |  |  |  |  |
|  | |  | | **Mean Difference** | | | | **SE** | | **t** | | **p _tukey_** |  |  |  |  |
| Brazil |  | Colombia |  | 10.457 | | |  | 1.778 |  | 5.882 |  | < .001 |  |  |  |  |
|  |  | Germany |  | 3.775 | | |  | 2.550 |  | 1.481 |  | 0.818 |  |  |  |  |
|  |  | Israel |  | 4.711 | | |  | 1.905 |  | 2.473 |  | 0.207 |  |  |  |  |
|  |  | Norway |  | 1.993 | | |  | 1.648 |  | 1.210 |  | 0.929 |  |  |  |  |
|  |  | US |  | -2.990 | | |  | 3.285 |  | -0.910 |  | 0.985 |  |  |  |  |
|  |  | empty |  | 3.921 | | |  | 1.828 |  | 2.145 |  | 0.386 |  |  |  |  |
|  |  | other |  | 2.699 | | |  | 2.558 |  | 1.055 |  | 0.966 |  |  |  |  |
| Colombia |  | Germany |  | -6.681 | | |  | 2.327 |  | -2.872 |  | 0.079 |  |  |  |  |
|  |  | Israel |  | -5.745 | | |  | 1.594 |  | -3.604 |  | 0.008 |  |  |  |  |
|  |  | Norway |  | -8.464 | | |  | 1.276 |  | -6.635 |  | < .001 |  |  |  |  |
|  |  | US |  | -13.447 | | |  | 3.115 |  | -4.317 |  | < .001 |  |  |  |  |
|  |  | empty |  | -6.536 | | |  | 1.501 |  | -4.353 |  | < .001 |  |  |  |  |
|  |  | other |  | -7.758 | | |  | 2.336 |  | -3.320 |  | 0.021 |  |  |  |  |
| Germany |  | Israel |  | 0.936 | | |  | 2.425 |  | 0.386 |  | 1.000 |  |  |  |  |
|  |  | Norway |  | -1.782 | | |  | 2.229 |  | -0.800 |  | 0.993 |  |  |  |  |
|  |  | US |  | -6.765 | | |  | 3.612 |  | -1.873 |  | 0.570 |  |  |  |  |
|  |  | empty |  | 0.146 | | |  | 2.365 |  | 0.062 |  | 1.000 |  |  |  |  |
|  |  | other |  | -1.076 | | |  | 2.966 |  | -0.363 |  | 1.000 |  |  |  |  |
| Israel |  | Norway |  | -2.718 | | |  | 1.447 |  | -1.878 |  | 0.566 |  |  |  |  |
|  |  | US |  | -7.701 | | |  | 3.189 |  | -2.415 |  | 0.234 |  |  |  |  |
|  |  | empty |  | -0.790 | | |  | 1.650 |  | -0.479 |  | 1.000 |  |  |  |  |
|  |  | other |  | -2.012 | | |  | 2.434 |  | -0.827 |  | 0.992 |  |  |  |  |
| Norway |  | US |  | -4.983 | | |  | 3.042 |  | -1.638 |  | 0.727 |  |  |  |  |
|  |  | empty |  | 1.928 | | |  | 1.344 |  | 1.434 |  | 0.841 |  |  |  |  |
|  |  | other |  | 0.706 | | |  | 2.239 |  | 0.315 |  | 1.000 |  |  |  |  |
| US |  | empty |  | 6.911 | | |  | 3.144 |  | 2.198 |  | 0.353 |  |  |  |  |
|  |  | other |  | 5.689 | | |  | 3.618 |  | 1.573 |  | 0.767 |  |  |  |  |
| empty |  | other |  | -1.222 | | |  | 2.375 |  | -0.515 |  | 1.000 |  |  |  |  |
|  | | | | | | | | | | | | |  |  |  |  |

| **Descriptives – Perceived Risk** | | | | | | | |
| --- | --- | --- | --- | --- | --- | --- | --- |
| **Countries** | | **Mean** | | **SD** | | **N** | |
| Brazil |  | 43.917 |  | 22.718 |  | 204 |  |
| Colombia |  | 33.460 |  | 20.814 |  | 418 |  |
| Germany |  | 40.141 |  | 19.324 |  | 99 |  |
| Israel |  | 39.205 |  | 20.214 |  | 288 |  |
| Norway |  | 41.924 |  | 20.512 |  | 734 |  |
| US |  | 46.907 |  | 22.197 |  | 50 |  |
| empty |  | 39.996 |  | 20.874 |  | 356 |  |
| other |  | 41.218 |  | 21.215 |  | 98 |  |
|  | | | | | | | |

| ANOVA - Knowledge per Country | | | | | | | | | | | | | | | | |
| --- | --- | --- | --- | --- | --- | --- | --- | --- | --- | --- | --- | --- | --- | --- | --- | --- |
| **Cases** | | | **Sum of Squares** | | **df** | | **Mean Square** | | | | **F** | | **p** | | **η²** | |
| Countries |  | | 121230.258 |  | 7.000 |  | 17318.608 | | |  | 31.733 |  | < .001 |  | 0.093 |  |
| Residual |  | | 1.180e +6 |  | 2162.000 |  | 545.758 | | |  |  |  |  |  |  |  |
| Note.  Type III Sum of Squares | | | | | | | | | | | | | | | | |
| **Post Hoc Comparisons - Countries** | | | | | | | | | | | | | |  |  |  |
|  | |  | | **Mean Difference** | | | | **SE** | | **t** | | **p _tukey_** | |  |  |  |
| Brazil |  | Colombia |  | 19.395 | | |  | 1.993 |  | 9.732 |  | < .001 |  |  |  |  |
|  |  | Germany |  | -2.587 | | |  | 2.871 |  | -0.901 |  | 0.986 |  |  |  |  |
|  |  | Israel |  | 3.933 | | |  | 2.149 |  | 1.830 |  | 0.599 |  |  |  |  |
|  |  | Norway |  | 3.034 | | |  | 1.852 |  | 1.638 |  | 0.727 |  |  |  |  |
|  |  | US |  | 0.430 | | |  | 3.687 |  | 0.117 |  | 1.000 |  |  |  |  |
|  |  | empty |  | -2.220 | | |  | 2.126 |  | -1.044 |  | 0.968 |  |  |  |  |
|  |  | other |  | -1.179 | | |  | 2.871 |  | -0.410 |  | 1.000 |  |  |  |  |
| Colombia |  | Germany |  | -21.982 | | |  | 2.620 |  | -8.389 |  | < .001 |  |  |  |  |
|  |  | Israel |  | -15.462 | | |  | 1.800 |  | -8.592 |  | < .001 |  |  |  |  |
|  |  | Norway |  | -16.361 | | |  | 1.433 |  | -11.421 |  | < .001 |  |  |  |  |
|  |  | US |  | -18.965 | | |  | 3.494 |  | -5.427 |  | < .001 |  |  |  |  |
|  |  | empty |  | -21.614 | | |  | 1.772 |  | -12.198 |  | < .001 |  |  |  |  |
|  |  | other |  | -20.573 | | |  | 2.620 |  | -7.852 |  | < .001 |  |  |  |  |
| Germany |  | Israel |  | 6.520 | | |  | 2.741 |  | 2.379 |  | 0.252 |  |  |  |  |
|  |  | Norway |  | 5.621 | | |  | 2.515 |  | 2.235 |  | 0.331 |  |  |  |  |
|  |  | US |  | 3.017 | | |  | 4.060 |  | 0.743 |  | 0.996 |  |  |  |  |
|  |  | empty |  | 0.367 | | |  | 2.723 |  | 0.135 |  | 1.000 |  |  |  |  |
|  |  | other |  | 1.408 | | |  | 3.337 |  | 0.422 |  | 1.000 |  |  |  |  |
| Israel |  | Norway |  | -0.899 | | |  | 1.643 |  | -0.548 |  | 0.999 |  |  |  |  |
|  |  | US |  | -3.503 | | |  | 3.586 |  | -0.977 |  | 0.978 |  |  |  |  |
|  |  | empty |  | -6.153 | | |  | 1.946 |  | -3.162 |  | 0.034 |  |  |  |  |
|  |  | other |  | -5.112 | | |  | 2.741 |  | -1.865 |  | 0.575 |  |  |  |  |
| Norway |  | US |  | -2.604 | | |  | 3.416 |  | -0.762 |  | 0.995 |  |  |  |  |
|  |  | empty |  | -5.254 | | |  | 1.612 |  | -3.258 |  | 0.025 |  |  |  |  |
|  |  | other |  | -4.213 | | |  | 2.515 |  | -1.675 |  | 0.704 |  |  |  |  |
| US |  | empty |  | -2.650 | | |  | 3.572 |  | -0.742 |  | 0.996 |  |  |  |  |
|  |  | other |  | -1.609 | | |  | 4.060 |  | -0.396 |  | 1.000 |  |  |  |  |
| empty |  | other |  | 1.041 | | |  | 2.723 |  | 0.382 |  | 1.000 |  |  |  |  |

| **Descriptives - Knowledge** | | | | | | | |
| --- | --- | --- | --- | --- | --- | --- | --- |
| **Countries** | | **Mean** | | **SD** | | **N** | |
| Brazil |  | 38.250 |  | 22.536 |  | 204 |  |
| Colombia |  | 18.855 |  | 28.254 |  | 421 |  |
| Germany |  | 40.837 |  | 21.995 |  | 98 |  |
| Israel |  | 34.317 |  | 25.488 |  | 281 |  |
| Norway |  | 35.216 |  | 20.877 |  | 722 |  |
| US |  | 37.820 |  | 19.671 |  | 50 |  |
| empty |  | 40.470 |  | 20.070 |  | 296 |  |
| other |  | 39.429 |  | 25.018 |  | 98 |  |

| ANOVA – Feeling of Control per Country | | | | | | | | | | | | | | | | |
| --- | --- | --- | --- | --- | --- | --- | --- | --- | --- | --- | --- | --- | --- | --- | --- | --- |
| **Cases** | | | **Sum of Squares** | | **df** | | **Mean Square** | | | | **F** | | **p** | | **η²** | |
| Countries |  | | 39585.644 |  | 7.000 |  | 5655.092 | | |  | 7.925 |  | < .001 |  | 0.031 |  |
| Residual |  | | 1.228e +6 |  | 1721.000 |  | 713.543 | | |  |  |  |  |  |  |  |
| Note.  Type III Sum of Squares | | | | | | | | | | | | | | | | |
| **Post Hoc Comparisons - Countries** | | | | | | | | | | | | | |  |  |  |
|  | |  | | **Mean Difference** | | | | **SE** | | **t** | | **p _tukey_** | |  |  |  |
| Brazil |  | Colombia |  | -2.332 | | |  | 2.290 |  | -1.018 |  | 0.972 |  |  |  |  |
|  |  | Germany |  | 17.113 | | |  | 3.669 |  | 4.665 |  | < .001 |  |  |  |  |
|  |  | Israel |  | 0.103 | | |  | 2.477 |  | 0.042 |  | 1.000 |  |  |  |  |
|  |  | Norway |  | 6.433 | | |  | 2.249 |  | 2.860 |  | 0.082 |  |  |  |  |
|  |  | US |  | 8.588 | | |  | 4.405 |  | 1.950 |  | 0.517 |  |  |  |  |
|  |  | empty |  | 5.228 | | |  | 2.850 |  | 1.834 |  | 0.597 |  |  |  |  |
|  |  | other |  | 7.056 | | |  | 3.456 |  | 2.042 |  | 0.454 |  |  |  |  |
| Colombia |  | Germany |  | 19.445 | | |  | 3.407 |  | 5.708 |  | < .001 |  |  |  |  |
|  |  | Israel |  | 2.435 | | |  | 2.069 |  | 1.177 |  | 0.938 |  |  |  |  |
|  |  | Norway |  | 8.765 | | |  | 1.790 |  | 4.897 |  | < .001 |  |  |  |  |
|  |  | US |  | 10.920 | | |  | 4.189 |  | 2.607 |  | 0.154 |  |  |  |  |
|  |  | empty |  | 7.560 | | |  | 2.504 |  | 3.019 |  | 0.052 |  |  |  |  |
|  |  | other |  | 9.388 | | |  | 3.176 |  | 2.956 |  | 0.063 |  |  |  |  |
| Germany |  | Israel |  | -17.010 | | |  | 3.535 |  | -4.812 |  | < .001 |  |  |  |  |
|  |  | Norway |  | -10.681 | | |  | 3.379 |  | -3.161 |  | 0.034 |  |  |  |  |
|  |  | US |  | -8.525 | | |  | 5.076 |  | -1.679 |  | 0.701 |  |  |  |  |
|  |  | empty |  | -11.886 | | |  | 3.806 |  | -3.123 |  | 0.038 |  |  |  |  |
|  |  | other |  | -10.057 | | |  | 4.278 |  | -2.351 |  | 0.267 |  |  |  |  |
| Israel |  | Norway |  | 6.330 | | |  | 2.023 |  | 3.128 |  | 0.038 |  |  |  |  |
|  |  | US |  | 8.485 | | |  | 4.294 |  | 1.976 |  | 0.499 |  |  |  |  |
|  |  | empty |  | 5.125 | | |  | 2.676 |  | 1.915 |  | 0.540 |  |  |  |  |
|  |  | other |  | 6.953 | | |  | 3.314 |  | 2.098 |  | 0.416 |  |  |  |  |
| Norway |  | US |  | 2.156 | | |  | 4.167 |  | 0.517 |  | 1.000 |  |  |  |  |
|  |  | empty |  | -1.205 | | |  | 2.466 |  | -0.489 |  | 1.000 |  |  |  |  |
|  |  | other |  | 0.624 | | |  | 3.147 |  | 0.198 |  | 1.000 |  |  |  |  |
| US |  | empty |  | -3.361 | | |  | 4.520 |  | -0.744 |  | 0.996 |  |  |  |  |
|  |  | other |  | -1.532 | | |  | 4.925 |  | -0.311 |  | 1.000 |  |  |  |  |
| empty |  | other |  | 1.829 | | |  | 3.601 |  | 0.508 |  | 1.000 |  |  |  |  |
|  | | | | | | | | | | | | | |  |  |  |

| **Descriptives – Feeling of Control** | | | | | | | |
| --- | --- | --- | --- | --- | --- | --- | --- |
| **Countries** | | **Mean** | | **SD** | | **N** | |
| Brazil |  | 57.433 |  | 28.986 |  | 201 |  |
| Colombia |  | 59.765 |  | 24.933 |  | 421 |  |
| Germany |  | 40.319 |  | 27.116 |  | 72 |  |
| Israel |  | 57.330 |  | 27.262 |  | 276 |  |
| Norway |  | 51.000 |  | 26.014 |  | 473 |  |
| US |  | 48.844 |  | 27.703 |  | 45 |  |
| empty |  | 52.205 |  | 27.620 |  | 156 |  |
| other |  | 50.376 |  | 29.044 |  | 85 |  |
|  | | | | | | | |

| ANOVA - worry/fear per Country | | | | | | | | | | | | | | | | |
| --- | --- | --- | --- | --- | --- | --- | --- | --- | --- | --- | --- | --- | --- | --- | --- | --- |
| **Cases** | | | **Sum of Squares** | | **df** | | **Mean Square** | | | | **F** | | **p** | | **η²** | |
| Countries | |  | 155985.980 |  | 7.000 |  | 22283.711 | | |  | 32.319 |  | < .001 |  | 0.115 |  |
| Residual | |  | 1.203e +6 |  | 1745.000 |  | 689.490 | | |  |  |  |  |  |  |  |
| Note.  Type III Sum of Squares | | | | | | | | | | | | | | | | |
| **Post Hoc Comparisons - Countries** | | | | | | | | | | | |  |  |  |  |  |
|  | |  | | **Mean Difference** | | **SE** | | **t** | | **p _tukey_** | |  |  |  |  |  |
| Brazil |  | Colombia |  | 15.733 |  | 2.244 |  | 7.012 |  | < .001 |  |  |  |  |  |  |
|  |  | Germany |  | 32.822 |  | 3.602 |  | 9.113 |  | < .001 |  |  |  |  |  |  |
|  |  | Israel |  | 17.887 |  | 2.412 |  | 7.417 |  | < .001 |  |  |  |  |  |  |
|  |  | Norway |  | 29.018 |  | 2.198 |  | 13.204 |  | < .001 |  |  |  |  |  |  |
|  |  | US |  | 12.225 |  | 4.214 |  | 2.901 |  | 0.073 |  |  |  |  |  |  |
|  |  | empty |  | 24.585 |  | 2.801 |  | 8.778 |  | < .001 |  |  |  |  |  |  |
|  |  | other |  | 29.960 |  | 3.351 |  | 8.940 |  | < .001 |  |  |  |  |  |  |
| Colombia |  | Germany |  | 17.090 |  | 3.349 |  | 5.103 |  | < .001 |  |  |  |  |  |  |
|  |  | Israel |  | 2.154 |  | 2.014 |  | 1.069 |  | 0.963 |  |  |  |  |  |  |
|  |  | Norway |  | 13.285 |  | 1.752 |  | 7.581 |  | < .001 |  |  |  |  |  |  |
|  |  | US |  | -3.508 |  | 4.000 |  | -0.877 |  | 0.988 |  |  |  |  |  |  |
|  |  | empty |  | 8.852 |  | 2.467 |  | 3.588 |  | 0.008 |  |  |  |  |  |  |
|  |  | other |  | 14.227 |  | 3.078 |  | 4.623 |  | < .001 |  |  |  |  |  |  |
| Germany |  | Israel |  | -14.936 |  | 3.463 |  | -4.312 |  | < .001 |  |  |  |  |  |  |
|  |  | Norway |  | -3.804 |  | 3.318 |  | -1.146 |  | 0.946 |  |  |  |  |  |  |
|  |  | US |  | -20.597 |  | 4.893 |  | -4.210 |  | < .001 |  |  |  |  |  |  |
|  |  | empty |  | -8.237 |  | 3.745 |  | -2.200 |  | 0.352 |  |  |  |  |  |  |
|  |  | other |  | -2.862 |  | 4.173 |  | -0.686 |  | 0.997 |  |  |  |  |  |  |
| Israel |  | Norway |  | 11.131 |  | 1.963 |  | 5.671 |  | < .001 |  |  |  |  |  |  |
|  |  | US |  | -5.662 |  | 4.097 |  | -1.382 |  | 0.866 |  |  |  |  |  |  |
|  |  | empty |  | 6.698 |  | 2.621 |  | 2.556 |  | 0.173 |  |  |  |  |  |  |
|  |  | other |  | 12.073 |  | 3.202 |  | 3.770 |  | 0.004 |  |  |  |  |  |  |
| Norway |  | US |  | -16.793 |  | 3.975 |  | -4.225 |  | < .001 |  |  |  |  |  |  |
|  |  | empty |  | -4.433 |  | 2.425 |  | -1.828 |  | 0.601 |  |  |  |  |  |  |
|  |  | other |  | 0.942 |  | 3.044 |  | 0.309 |  | 1.000 |  |  |  |  |  |  |
| US |  | empty |  | 12.360 |  | 4.337 |  | 2.850 |  | 0.084 |  |  |  |  |  |  |
|  |  | other |  | 17.735 |  | 4.712 |  | 3.764 |  | 0.004 |  |  |  |  |  |  |
| empty |  | other |  | 5.375 |  | 3.505 |  | 1.534 |  | 0.789 |  |  |  |  |  |  |
|  | | | | | | | | | | | |  |  |  |  |  |

| **Descriptives - worry/fear** | | | | | | | |
| --- | --- | --- | --- | --- | --- | --- | --- |
| **Countries** | | **Mean** | | **SD** | | **N** | |
| Brazil |  | 79.204 |  | 22.035 |  | 203 |  |
| Colombia |  | 63.471 |  | 26.140 |  | 421 |  |
| Germany |  | 46.382 |  | 25.254 |  | 72 |  |
| Israel |  | 61.318 |  | 27.392 |  | 285 |  |
| Norway |  | 50.186 |  | 26.612 |  | 481 |  |
| US |  | 66.979 |  | 24.787 |  | 48 |  |
| empty |  | 54.619 |  | 28.093 |  | 155 |  |
| other |  | 49.244 |  | 28.237 |  | 88 |  |
|  | | | | | | | |

| ANOVA - CORE-9 / general distress per Country | | | | | | | | | | | | | | | | |
| --- | --- | --- | --- | --- | --- | --- | --- | --- | --- | --- | --- | --- | --- | --- | --- | --- |
| **Cases** | | | **Sum of Squares** | | **df** | | **Mean Square** | | | | **F** | | **p** | | **η²** | |
| Countries | |  | 200.527 |  | 7.000 |  | 28.647 | | |  | 67.377 |  | < .001 |  | 0.178 |  |
| Residual | |  | 923.046 |  | 2171.000 |  | 0.425 | | |  |  |  |  |  |  |  |
| Note.  Type III Sum of Squares | | | | | | | | | | | | | | | | |
| **Post Hoc Comparisons - Countries** | | | | | | | | | | | |  |  |  |  |  |
|  | |  | | **Mean Difference** | | **SE** | | **t** | | **p _tukey_** | |  |  |  |  |  |
| Brazil |  | Colombia |  | -0.298 |  | 0.056 |  | -5.364 |  | < .001 |  |  |  |  |  |  |
|  |  | Germany |  | 0.410 |  | 0.080 |  | 5.140 |  | < .001 |  |  |  |  |  |  |
|  |  | Israel |  | 0.149 |  | 0.060 |  | 2.476 |  | 0.206 |  |  |  |  |  |  |
|  |  | Norway |  | 0.488 |  | 0.052 |  | 9.451 |  | < .001 |  |  |  |  |  |  |
|  |  | US |  | 0.037 |  | 0.103 |  | 0.356 |  | 1.000 |  |  |  |  |  |  |
|  |  | empty |  | 0.471 |  | 0.060 |  | 7.903 |  | < .001 |  |  |  |  |  |  |
|  |  | other |  | 0.184 |  | 0.080 |  | 2.301 |  | 0.293 |  |  |  |  |  |  |
| Colombia |  | Germany |  | 0.709 |  | 0.073 |  | 9.732 |  | < .001 |  |  |  |  |  |  |
|  |  | Israel |  | 0.447 |  | 0.050 |  | 8.895 |  | < .001 |  |  |  |  |  |  |
|  |  | Norway |  | 0.786 |  | 0.040 |  | 19.711 |  | < .001 |  |  |  |  |  |  |
|  |  | US |  | 0.335 |  | 0.098 |  | 3.435 |  | 0.014 |  |  |  |  |  |  |
|  |  | empty |  | 0.769 |  | 0.050 |  | 15.463 |  | < .001 |  |  |  |  |  |  |
|  |  | other |  | 0.482 |  | 0.073 |  | 6.620 |  | < .001 |  |  |  |  |  |  |
| Germany |  | Israel |  | -0.262 |  | 0.076 |  | -3.438 |  | 0.014 |  |  |  |  |  |  |
|  |  | Norway |  | 0.077 |  | 0.070 |  | 1.106 |  | 0.956 |  |  |  |  |  |  |
|  |  | US |  | -0.374 |  | 0.113 |  | -3.305 |  | 0.022 |  |  |  |  |  |  |
|  |  | empty |  | 0.060 |  | 0.076 |  | 0.792 |  | 0.994 |  |  |  |  |  |  |
|  |  | other |  | -0.227 |  | 0.093 |  | -2.446 |  | 0.220 |  |  |  |  |  |  |
| Israel |  | Norway |  | 0.339 |  | 0.046 |  | 7.416 |  | < .001 |  |  |  |  |  |  |
|  |  | US |  | -0.112 |  | 0.100 |  | -1.118 |  | 0.953 |  |  |  |  |  |  |
|  |  | empty |  | 0.322 |  | 0.055 |  | 5.905 |  | < .001 |  |  |  |  |  |  |
|  |  | other |  | 0.035 |  | 0.076 |  | 0.463 |  | 1.000 |  |  |  |  |  |  |
| Norway |  | US |  | -0.451 |  | 0.095 |  | -4.733 |  | < .001 |  |  |  |  |  |  |
|  |  | empty |  | -0.017 |  | 0.045 |  | -0.379 |  | 1.000 |  |  |  |  |  |  |
|  |  | other |  | -0.304 |  | 0.070 |  | -4.353 |  | < .001 |  |  |  |  |  |  |
| US |  | empty |  | 0.434 |  | 0.100 |  | 4.347 |  | < .001 |  |  |  |  |  |  |
|  |  | other |  | 0.147 |  | 0.113 |  | 1.301 |  | 0.899 |  |  |  |  |  |  |
| empty |  | other |  | -0.287 |  | 0.076 |  | -3.780 |  | 0.004 |  |  |  |  |  |  |
|  | | | | | | | | | | | |  |  |  |  |  |

| **Descriptives - CORE-9 / general distress** | | | | | | | |
| --- | --- | --- | --- | --- | --- | --- | --- |
| **Countries** | | **Mean** | | **SD** | | **N** | |
| Brazil |  | 1.248 |  | 0.782 |  | 204 |  |
| Colombia |  | 1.546 |  | 0.755 |  | 420 |  |
| Germany |  | 0.837 |  | 0.577 |  | 99 |  |
| Israel |  | 1.099 |  | 0.622 |  | 281 |  |
| Norway |  | 0.760 |  | 0.548 |  | 735 |  |
| US |  | 1.211 |  | 0.867 |  | 50 |  |
| empty |  | 0.777 |  | 0.610 |  | 291 |  |
| other |  | 1.064 |  | 0.740 |  | 99 |  |
|  | | | | | | | |

| ANOVA - CAPE-P / paranoia per Country | | | | | | | | | | | | | | | | |
| --- | --- | --- | --- | --- | --- | --- | --- | --- | --- | --- | --- | --- | --- | --- | --- | --- |
| **Cases** | | | **Sum of Squares** | | **df** | | **Mean Square** | | | | **F** | | **p** | | **η²** | |
| Countries | |  | 53.461 |  | 7.000 |  | 7.637 | | |  | 90.544 |  | < .001 |  | 0.221 |  |
| Residual | |  | 188.014 |  | 2229.000 |  | 0.084 | | |  |  |  |  |  |  |  |
| Note.  Type III Sum of Squares | | | | | | | | | | | | | | | | |
| **Post Hoc Comparisons - Countries** | | | | | | | | | | | |  |  |  |  |  |
|  | |  | | **Mean Difference** | | **SE** | | **t** | | **p _tukey_** | |  |  |  |  |  |
| Brazil |  | Colombia |  | -0.168 |  | 0.025 |  | -6.776 |  | < .001 |  |  |  |  |  |  |
|  |  | Germany |  | 0.098 |  | 0.036 |  | 2.752 |  | 0.108 |  |  |  |  |  |  |
|  |  | Israel |  | 0.033 |  | 0.027 |  | 1.230 |  | 0.923 |  |  |  |  |  |  |
|  |  | Norway |  | 0.249 |  | 0.023 |  | 10.822 |  | < .001 |  |  |  |  |  |  |
|  |  | US |  | 0.122 |  | 0.046 |  | 2.662 |  | 0.135 |  |  |  |  |  |  |
|  |  | empty |  | 0.203 |  | 0.026 |  | 7.886 |  | < .001 |  |  |  |  |  |  |
|  |  | other |  | 0.120 |  | 0.035 |  | 3.384 |  | 0.017 |  |  |  |  |  |  |
| Colombia |  | Germany |  | 0.266 |  | 0.032 |  | 8.193 |  | < .001 |  |  |  |  |  |  |
|  |  | Israel |  | 0.201 |  | 0.022 |  | 9.040 |  | < .001 |  |  |  |  |  |  |
|  |  | Norway |  | 0.417 |  | 0.018 |  | 23.469 |  | < .001 |  |  |  |  |  |  |
|  |  | US |  | 0.290 |  | 0.043 |  | 6.672 |  | < .001 |  |  |  |  |  |  |
|  |  | empty |  | 0.371 |  | 0.021 |  | 17.497 |  | < .001 |  |  |  |  |  |  |
|  |  | other |  | 0.288 |  | 0.032 |  | 8.910 |  | < .001 |  |  |  |  |  |  |
| Germany |  | Israel |  | -0.065 |  | 0.034 |  | -1.928 |  | 0.531 |  |  |  |  |  |  |
|  |  | Norway |  | 0.151 |  | 0.031 |  | 4.851 |  | < .001 |  |  |  |  |  |  |
|  |  | US |  | 0.024 |  | 0.050 |  | 0.478 |  | 1.000 |  |  |  |  |  |  |
|  |  | empty |  | 0.105 |  | 0.033 |  | 3.166 |  | 0.034 |  |  |  |  |  |  |
|  |  | other |  | 0.022 |  | 0.041 |  | 0.536 |  | 0.999 |  |  |  |  |  |  |
| Israel |  | Norway |  | 0.216 |  | 0.020 |  | 10.714 |  | < .001 |  |  |  |  |  |  |
|  |  | US |  | 0.089 |  | 0.044 |  | 2.008 |  | 0.477 |  |  |  |  |  |  |
|  |  | empty |  | 0.170 |  | 0.023 |  | 7.323 |  | < .001 |  |  |  |  |  |  |
|  |  | other |  | 0.087 |  | 0.034 |  | 2.591 |  | 0.160 |  |  |  |  |  |  |
| Norway |  | US |  | -0.127 |  | 0.042 |  | -2.986 |  | 0.057 |  |  |  |  |  |  |
|  |  | empty |  | -0.046 |  | 0.019 |  | -2.401 |  | 0.241 |  |  |  |  |  |  |
|  |  | other |  | -0.129 |  | 0.031 |  | -4.159 |  | < .001 |  |  |  |  |  |  |
| US |  | empty |  | 0.081 |  | 0.044 |  | 1.840 |  | 0.593 |  |  |  |  |  |  |
|  |  | other |  | -0.002 |  | 0.050 |  | -0.040 |  | 1.000 |  |  |  |  |  |  |
| empty |  | other |  | -0.083 |  | 0.033 |  | -2.510 |  | 0.191 |  |  |  |  |  |  |
|  | | | | | | | | | | | |  |  |  |  |  |

| **Descriptives - CAPE-P** | | | | | | | |
| --- | --- | --- | --- | --- | --- | --- | --- |
| **Countries** | | **Mean** | | **SD** | | **N** | |
| Brazil |  | 1.532 |  | 0.354 |  | 204 |  |
| Colombia |  | 1.700 |  | 0.386 |  | 421 |  |
| Germany |  | 1.434 |  | 0.259 |  | 99 |  |
| Israel |  | 1.499 |  | 0.291 |  | 289 |  |
| Norway |  | 1.283 |  | 0.219 |  | 735 |  |
| US |  | 1.410 |  | 0.276 |  | 50 |  |
| empty |  | 1.329 |  | 0.254 |  | 339 |  |
| other |  | 1.412 |  | 0.289 |  | 100 |  |
|  | | | | | | | |

### Relationship between restrictions and reactions per country


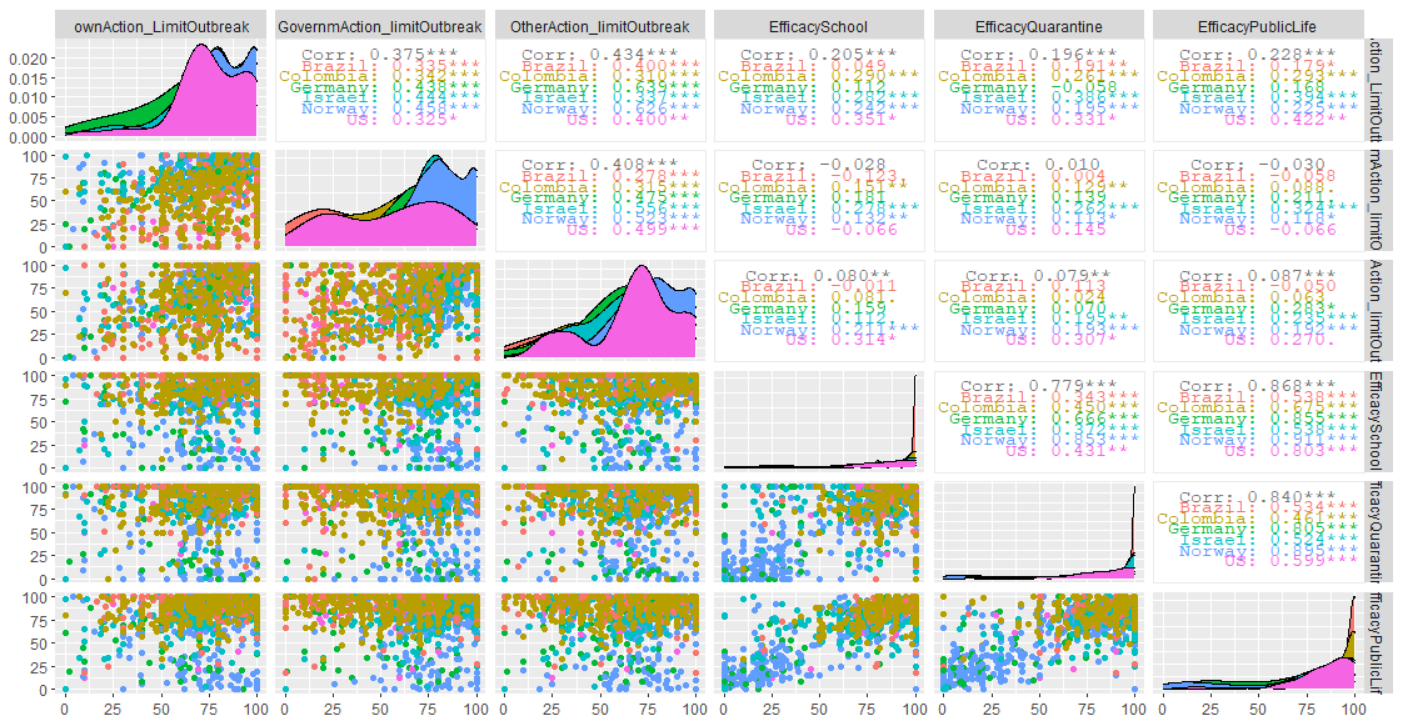


*Fig S2: Correlation plot for the perceived efficacy of own, other or governmental actions and efficacy of countermeasures such as school closings, quarantine and public life restrictions per country*

### Hypothesis 1: Direct impact of restrictions on daily life

Those that stated not being personally affected, had to have zero in the “yes” column

|  | | | | | | | | | |
| --- | --- | --- | --- | --- | --- | --- | --- | --- | --- |
|  | | | | **School closings** | | | |  | |
| **Personally affected** | | **Severity on Daily Life** | | **no** | | **yes** | | **Total** | |
| no |  | Not much |  | 152 |  | 0 |  | 152 |  |
|  |  | Somewhat |  | 125 |  | 0 |  | 125 |  |
|  |  | Very much |  | 154 |  | 0 |  | 154 |  |
|  |  | Total |  | 431 |  | 0 |  | 431 |  |
| yes |  | Not much |  | 410 |  | 28 |  | 438 |  |
|  |  | Somewhat |  | 288 |  | 111 |  | 399 |  |
|  |  | Very much |  | 530 |  | 463 |  | 993 |  |
|  |  | Total |  | 1228 |  | 602 |  | 1830 |  |
| Total |  | Not much |  | 562 |  | 28 |  | 590 |  |
|  |  | Somewhat |  | 413 |  | 111 |  | 524 |  |
|  |  | Very much |  | 684 |  | 463 |  | 1147 |  |
|  |  | Total |  | 1659 |  | 602 |  | 2261 |  |
|  | | | | | | | | | |

| **Chi-Squared Tests** | | | | | | | | | |  |  |  |  |  |  |
| --- | --- | --- | --- | --- | --- | --- | --- | --- | --- | --- | --- | --- | --- | --- | --- |
| **Personally affected** | |  | | **Value** | | **df** | | **p** | |  |  |  |  |  |  |
| yes |  | Χ² |  | 228.837 |  | 2 |  | < .001 |  |  |  |  |  |  |  |
|  |  | N |  | 1830 |  |  |  |  |  |  |  |  |  |  |  |
| Total |  | Χ² |  | 263.370 |  | 2 |  | < .001 |  |  |  |  |  |  |  |
|  |  | N |  | 2261 |  |  |  |  |  |  |  |  |  |  |  |
|  | | | | | | | | | |  |  |  |  |  |  |
|  | | | | | | | | | |  |  |  |  |  |  |
|  | | | | | | | | | | | | | | | |
|  | | | | | | | | | | **Transport** | | | |  | |
| **Personally affected** | | **Severity on DailyLife** | | | | | | | | **no** | | **yes** | | **Total** | |
| no |  | Not much | | | | | | |  | 161 |  | 0 |  | 161 |  |
|  |  | Somewhat | | | | | | |  | 184 |  | 0 |  | 184 |  |
|  |  | Very much | | | | | | |  | 86 |  | 0 |  | 86 |  |
|  |  | Total | | | | | | |  | 431 |  | 0 |  | 431 |  |
| yes |  | Not much | | | | | | |  | 592 |  | 87 |  | 679 |  |
|  |  | Somewhat | | | | | | |  | 551 |  | 286 |  | 837 |  |
|  |  | Very much | | | | | | |  | 143 |  | 170 |  | 313 |  |
|  |  | Total | | | | | | |  | 1286 |  | 543 |  | 1829 |  |
| Total |  | Not much | | | | | | |  | 753 |  | 87 |  | 840 |  |
|  |  | Somewhat | | | | | | |  | 735 |  | 286 |  | 1021 |  |
|  |  | Very much | | | | | | |  | 229 |  | 170 |  | 399 |  |
|  |  | Total | | | | | | |  | 1717 |  | 543 |  | 2260 |  |
|  | | | | | | | | | | | | | | | |

| **Chi-Squared Tests** | | | | | | | | | |  |  |  |  |  |  |  |
| --- | --- | --- | --- | --- | --- | --- | --- | --- | --- | --- | --- | --- | --- | --- | --- | --- |
| **Personally affected** | |  | | **Value** | | **df** | | **p** | |  |  |  |  |  |  |  |
| yes |  | Χ² |  | 191.608 |  | 2 |  | < .001 |  |  |  |  |  |  |  |  |
|  |  | N |  | 1829 |  |  |  |  |  |  |  |  |  |  |  |  |
| Total |  | Χ² |  | 170.328 |  | 2 |  | < .001 |  |  |  |  |  |  |  |  |
|  |  | N |  | 2260 |  |  |  |  |  |  |  |  |  |  |  |  |
|  | | | | | | | | | |  |  |  |  |  |  |  |
|  | | | | | | | | | |  |  |  |  |  |  |  |
|  | | | | | | | | | | | | | | | | |
|  | | | | | | | | | | | **Quarantine** | | | |  | |
| **Personally affected** | | **Severity for daily Life** | | | | | | | | | **no** | | **yes** | | **Total** | |
| no |  | Not much | | | | | | | |  | 102 |  | 0 |  | 102 |  |
|  |  | Somewhat | | | | | | | |  | 167 |  | 0 |  | 167 |  |
|  |  | Very much | | | | | | | |  | 162 |  | 0 |  | 162 |  |
|  |  | Total | | | | | | | |  | 431 |  | 0 |  | 431 |  |
| yes |  | Not much | | | | | | | |  | 240 |  | 69 |  | 309 |  |
|  |  | Somewhat | | | | | | | |  | 434 |  | 217 |  | 651 |  |
|  |  | Very much | | | | | | | |  | 528 |  | 342 |  | 870 |  |
|  |  | Total | | | | | | | |  | 1202 |  | 628 |  | 1830 |  |
| Total |  | Not much | | | | | | | |  | 342 |  | 69 |  | 411 |  |
|  |  | Somewhat | | | | | | | |  | 601 |  | 217 |  | 818 |  |
|  |  | Very much | | | | | | | |  | 690 |  | 342 |  | 1032 |  |
|  |  | Total | | | | | | | |  | 1633 |  | 628 |  | 2261 |  |
|  | | | | | | | | | | | | | | | | |

| **Chi-Squared Tests** | | | | | | | | | |  |  |  |  |  |  |  |
| --- | --- | --- | --- | --- | --- | --- | --- | --- | --- | --- | --- | --- | --- | --- | --- | --- |
| **Personally affected** | |  | | **Value** | | **df** | | **p** | |  |  |  |  |  |  |  |
| yes |  | Χ² |  | 29.601 |  | 2 |  | < .001 |  |  |  |  |  |  |  |  |
|  |  | N |  | 1830 |  |  |  |  |  |  |  |  |  |  |  |  |
| Total |  | Χ² |  | 40.169 |  | 2 |  | < .001 |  |  |  |  |  |  |  |  |
|  |  | N |  | 2261 |  |  |  |  |  |  |  |  |  |  |  |  |
|  | | | | | | | | | |  |  |  |  |  |  |  |
|  | | | | | | | | | |  |  |  |  |  |  |  |
|  | | | | | | | | | | | | | | | | |
|  | | | | | | | | | | | **Sport & Culture** | | | |  | |
| **Personally affected** | | **Severity for daily Life** | | | | | | | | | **no** | | **yes** | | **Total** | |
| no |  | Not much | | | | | | | |  | 155 |  | 0 |  | 155 |  |
|  |  | Somewhat | | | | | | | |  | 166 |  | 0 |  | 166 |  |
|  |  | Very much | | | | | | | |  | 110 |  | 0 |  | 110 |  |
|  |  | Total | | | | | | | |  | 431 |  | 0 |  | 431 |  |
| yes |  | Not much | | | | | | | |  | 435 |  | 96 |  | 531 |  |
|  |  | Somewhat | | | | | | | |  | 386 |  | 444 |  | 830 |  |
|  |  | Very much | | | | | | | |  | 103 |  | 366 |  | 469 |  |
|  |  | Total | | | | | | | |  | 924 |  | 906 |  | 1830 |  |
| Total |  | Not much | | | | | | | |  | 590 |  | 96 |  | 686 |  |
|  |  | Somewhat | | | | | | | |  | 552 |  | 444 |  | 996 |  |
|  |  | Very much | | | | | | | |  | 213 |  | 366 |  | 579 |  |
|  |  | Total | | | | | | | |  | 1355 |  | 906 |  | 2261 |  |
|  | | | | | | | | | | | | | | | | |

| **Chi-Squared Tests** | | | | | | | | | |  |  |  |  |  |  |  |
| --- | --- | --- | --- | --- | --- | --- | --- | --- | --- | --- | --- | --- | --- | --- | --- | --- |
| **Personally affected** | |  | | **Value** | | **df** | | **p** | |  |  |  |  |  |  |  |
| yes |  | Χ² |  | 367.817 |  | 2 |  | < .001 |  |  |  |  |  |  |  |  |
|  |  | N |  | 1830 |  |  |  |  |  |  |  |  |  |  |  |  |
| Total |  | Χ² |  | 331.799 |  | 2 |  | < .001 |  |  |  |  |  |  |  |  |
|  |  | N |  | 2261 |  |  |  |  |  |  |  |  |  |  |  |  |
|  | | | | | | | | | |  |  |  |  |  |  |  |
|  | | | | | | | | | |  |  |  |  |  |  |  |
|  | | | | | | | | | | | | | | | | |
|  | | | | | | | | | | | **Travel ban** | | | |  | |
| **Personally affected** | | **Severity for daily Life** | | | | | | | | | **no** | | **yes** | | **Total** | |
| no |  | Not much | | | | | | | |  | 167 |  | 0 |  | 167 |  |
|  |  | Somewhat | | | | | | | |  | 162 |  | 0 |  | 162 |  |
|  |  | Very much | | | | | | | |  | 102 |  | 0 |  | 102 |  |
|  |  | Total | | | | | | | |  | 431 |  | 0 |  | 431 |  |
| yes |  | Not much | | | | | | | |  | 639 |  | 175 |  | 814 |  |
|  |  | Somewhat | | | | | | | |  | 341 |  | 293 |  | 634 |  |
|  |  | Very much | | | | | | | |  | 162 |  | 219 |  | 381 |  |
|  |  | Total | | | | | | | |  | 1142 |  | 687 |  | 1829 |  |
| Total |  | Not much | | | | | | | |  | 806 |  | 175 |  | 981 |  |
|  |  | Somewhat | | | | | | | |  | 503 |  | 293 |  | 796 |  |
|  |  | Very much | | | | | | | |  | 264 |  | 219 |  | 483 |  |
|  |  | Total | | | | | | | |  | 1573 |  | 687 |  | 2260 |  |
|  | | | | | | | | | | | | | | | | |

| **Chi-Squared Tests** | | | | | | | | | |  |  |  |  |  |  |  |
| --- | --- | --- | --- | --- | --- | --- | --- | --- | --- | --- | --- | --- | --- | --- | --- | --- |
| **Personally affected** | |  | | **Value** | | **df** | | **p** | |  |  |  |  |  |  |  |
| yes |  | Χ² |  | 174.246 |  | 2 |  | < .001 |  |  |  |  |  |  |  |  |
|  |  | N |  | 1829 |  |  |  |  |  |  |  |  |  |  |  |  |
| Total |  | Χ² |  | 139.575 |  | 2 |  | < .001 |  |  |  |  |  |  |  |  |
|  |  | N |  | 2260 |  |  |  |  |  |  |  |  |  |  |  |  |
|  | | | | | | | | | |  |  |  |  |  |  |  |
|  | | | | | | | | | |  |  |  |  |  |  |  |
|  | | | | | | | | | | | | | | | | |
|  | | | | | | | | | | | **Social Distancing** | | | |  | |
| **Personally affected** | | **Severity for daily Life** | | | | | | | | | **no** | | **yes** | | **Total** | |
| no |  | Not much | | | | | | | |  | 86 |  | 0 |  | 86 |  |
|  |  | Somewhat | | | | | | | |  | 195 |  | 0 |  | 195 |  |
|  |  | Very much | | | | | | | |  | 150 |  | 0 |  | 150 |  |
|  |  | Total | | | | | | | |  | 431 |  | 0 |  | 431 |  |
| yes |  | Not much | | | | | | | |  | 202 |  | 68 |  | 270 |  |
|  |  | Somewhat | | | | | | | |  | 445 |  | 351 |  | 796 |  |
|  |  | Very much | | | | | | | |  | 333 |  | 431 |  | 764 |  |
|  |  | Total | | | | | | | |  | 980 |  | 850 |  | 1830 |  |
| Total |  | Not much | | | | | | | |  | 288 |  | 68 |  | 356 |  |
|  |  | Somewhat | | | | | | | |  | 640 |  | 351 |  | 991 |  |
|  |  | Very much | | | | | | | |  | 483 |  | 431 |  | 914 |  |
|  |  | Total | | | | | | | |  | 1411 |  | 850 |  | 2261 |  |
|  | | | | | | | | | | | | | | | | |

| **Chi-Squared Tests** | | | | | | | | | |
| --- | --- | --- | --- | --- | --- | --- | --- | --- | --- |
| **Personally affected** | |  | | **Value** | | **df** | | **p** | |
| yes |  | Χ² |  | 81.350 |  | 2 |  | < .001 |  |
|  |  | N |  | 1830 |  |  |  |  |  |
| Total |  | Χ² |  | 89.508 |  | 2 |  | < .001 |  |
|  |  | N |  | 2261 |  |  |  |  |  |
|  | | | | | | | | | |
|  | | | | | | | | | |

### Hypothesis 2: How do participants perceive efficacy of their own, other, and governmental reactions?

Post-hoc test for perceived efficacy of own / governmental / other reaction and per country

| **Post Hoc Comparisons -** own / governmental / other reaction | | | | | | | | | | | | | | | | | |
| --- | --- | --- | --- | --- | --- | --- | --- | --- | --- | --- | --- | --- | --- | --- | --- | --- | --- |
|  | | | | | | **95% CI of Mean Difference** | | | |  | | | | | | | |
|  | |  | | **Mean Difference** | | **Lower** | | **Upper** | | **SE** | | **t** | | **Cohen's d** | | **p _holm_** | |
| government |  | other |  | -0.204 |  | -1.953 |  | 1.546 |  | 0.730 |  | -0.279 |  | -0.007 |  | 0.780 |  |
|  |  | own |  | -7.850 |  | -9.520 |  | -6.181 |  | 0.697 |  | -11.269 |  | -0.295 |  | < .001 |  |
| other |  | own |  | -7.647 |  | -9.208 |  | -6.085 |  | 0.651 |  | -11.737 |  | -0.307 |  | < .001 |  |
|  | | | | | | | | | | | | | | | | | |
| *Note.*  Cohen's d does not correct for multiple comparisons. | | | | | | | | | | | | | | | | | |
| *Note.*  Bonferroni adjusted confidence intervals. | | | | | | | | | | | | | | | | | |

| **Post Hoc Comparisons - Countries** | | | | | | | | | | | | | | | | | |
| --- | --- | --- | --- | --- | --- | --- | --- | --- | --- | --- | --- | --- | --- | --- | --- | --- | --- |
|  | | | | | | **95% CI of Mean Difference** | | | |  | | | | | | | |
|  | |  | | **Mean Difference** | | **Lower** | | **Upper** | | **SE** | | **t** | | **Cohen's d** | | **p _holm_** | |
| Brazil |  | Colombia |  | -1.195 |  | -7.038 |  | 4.647 |  | 1.987 |  | -0.602 |  | -0.016 |  | 1.000 |  |
|  |  | Germany |  | 5.064 |  | -3.358 |  | 13.485 |  | 2.864 |  | 1.768 |  | 0.046 |  | 0.464 |  |
|  |  | Israel |  | -7.165 |  | -13.375 |  | -0.956 |  | 2.112 |  | -3.393 |  | -0.089 |  | 0.006 |  |
|  |  | Norway |  | -12.487 |  | -18.251 |  | -6.722 |  | 1.961 |  | -6.369 |  | -0.166 |  | < .001 |  |
|  |  | US |  | -1.649 |  | -14.197 |  | 10.898 |  | 4.268 |  | -0.386 |  | -0.010 |  | 1.000 |  |
| Colombia |  | Germany |  | 6.259 |  | -0.862 |  | 13.380 |  | 2.422 |  | 2.584 |  | 0.068 |  | 0.069 |  |
|  |  | Israel |  | -5.970 |  | -10.253 |  | -1.687 |  | 1.457 |  | -4.098 |  | -0.107 |  | < .001 |  |
|  |  | Norway |  | -11.291 |  | -14.900 |  | -7.683 |  | 1.227 |  | -9.200 |  | -0.240 |  | < .001 |  |
|  |  | US |  | -0.454 |  | -12.168 |  | 11.260 |  | 3.984 |  | -0.114 |  | -0.003 |  | 1.000 |  |
| Germany |  | Israel |  | -12.229 |  | -19.654 |  | -4.804 |  | 2.525 |  | -4.842 |  | -0.127 |  | < .001 |  |
|  |  | Norway |  | -17.550 |  | -24.607 |  | -10.493 |  | 2.400 |  | -7.312 |  | -0.191 |  | < .001 |  |
|  |  | US |  | -6.713 |  | -19.904 |  | 6.478 |  | 4.487 |  | -1.496 |  | -0.039 |  | 0.674 |  |
| Israel |  | Norway |  | -5.321 |  | -9.498 |  | -1.145 |  | 1.421 |  | -3.746 |  | -0.098 |  | 0.002 |  |
|  |  | US |  | 5.516 |  | -6.386 |  | 17.418 |  | 4.048 |  | 1.363 |  | 0.036 |  | 0.693 |  |
| Norway |  | US |  | 10.837 |  | -0.838 |  | 22.513 |  | 3.971 |  | 2.729 |  | 0.071 |  | 0.051 |  |
|  | | | | | | | | | | | | | | | | | |
| *Note.*  Cohen's d does not correct for multiple comparisons. | | | | | | | | | | | | | | | | | |
| *Note.*  Bonferroni adjusted confidence intervals. | | | | | | | | | | | | | | | | | |

## Linear Regression

For all participants (no restriction to the six countries), Average perceived efficacy of actions explained 14.8% of the variance. Adding the stringency index, we found that the model improved by .1%.

| **Model Summary** | | | | | | | | | | | | | | | | |
| --- | --- | --- | --- | --- | --- | --- | --- | --- | --- | --- | --- | --- | --- | --- | --- | --- |
| **Model** | | | **R** | | **R²** | | **Adjusted R²** | | | | | | **RMSE** | | | |
| 0 | |  | 0.385 |  | 0.148 |  | 0.141 | | | | |  | 17.774 | |  | |
| 1 | |  | 0.385 |  | 0.149 |  | 0.141 | | | | |  | 17.775 | |  | |
| Note.  Null model includes Knowledge, Feeling of Control, Perceived Risk, Number of Actions, CORE-9, worry/fear, Gender, CAPE-P, Age | | | | | | | | | | | | | | | | |
| **ANOVA** | | | | | | | | | | | | | | | | |
| **Model** | |  | | **Sum of Squares** | | | | **df** | | **Mean Square** | | **F** | | **p** | | |
| 0 |  | Regression |  | 62771.503 | | |  | 9 |  | 6974.611 |  | 22.077 |  | < .001 | |  |
|  |  | Residual |  | 361731.913 | | |  | 1145 |  | 315.923 |  |  |  |  | |  |
|  |  | Total |  | 424503.416 | | |  | 1154 |  |  |  |  |  |  | |  |
| 1 |  | Regression |  | 63071.655 | | |  | 10 |  | 6307.165 |  | 19.963 |  | < .001 | |  |
|  |  | Residual |  | 361431.761 | | |  | 1144 |  | 315.937 |  |  |  |  | |  |
|  |  | Total |  | 424503.416 | | |  | 1154 |  |  |  |  |  |  | |  |
| Note.  Null model includes Knowledge, FeelingControl, PercRisk, NumbActions, CORE-9, worry/fear, Gender, CAPE-P, age | | | | | | | | | | | | | | | | |

| **Coefficients** | | | | | | | | | | | | | | | | | |
| --- | --- | --- | --- | --- | --- | --- | --- | --- | --- | --- | --- | --- | --- | --- | --- | --- | --- |
|  | | | | | | | | | | | | | | **95% CI** | | | |
| **M** | |  | | **Unstandardized** | | **Standard Error** | | **Standardized** | | **t** | | **p** | | **Lower** | | **Upper** | |
| 0 |  | (Intercept) |  | 58.882 |  | 4.199 |  |  |  | 14.022 |  | < .001 |  | 50.643 |  | 67.122 |  |
|  |  | Knowledge |  | 0.019 |  | 0.021 |  | 0.026 |  | 0.929 |  | 0.353 |  | -0.021 |  | 0.060 |  |
|  |  | Feeling of Control |  | 0.207 |  | 0.020 |  | 0.296 |  | 10.627 |  | < .001 |  | 0.169 |  | 0.246 |  |
|  |  | Perceived Risk |  | 0.049 |  | 0.025 |  | 0.056 |  | 1.930 |  | 0.054 |  | -8.232e -4 |  | 0.099 |  |
|  |  | Number of Actions |  | 1.857 |  | 0.646 |  | 0.080 |  | 2.874 |  | 0.004 |  | 0.589 |  | 3.124 |  |
|  |  | General Distress |  | -3.398 |  | 0.877 |  | -0.133 |  | -3.877 |  | < .001 |  | -5.118 |  | -1.678 |  |
|  |  | Worry/Fear |  | 0.018 |  | 0.022 |  | 0.026 |  | 0.811 |  | 0.418 |  | -0.025 |  | 0.061 |  |
|  |  | Gender |  | 2.135 |  | 1.138 |  | 0.052 |  | 1.877 |  | 0.061 |  | -0.097 |  | 4.367 |  |
|  |  | Paranoia |  | -5.824 |  | 1.734 |  | -0.109 |  | -3.359 |  | < .001 |  | -9.225 |  | -2.422 |  |
|  |  | Age |  | 0.037 |  | 0.046 |  | 0.024 |  | 0.809 |  | 0.418 |  | -0.053 |  | 0.128 |  |
| 1 |  | (Intercept) |  | 60.507 |  | 4.518 |  |  |  | 13.392 |  | < .001 |  | 51.642 |  | 69.372 |  |
|  |  | Knowledge |  | 0.018 |  | 0.021 |  | 0.025 |  | 0.891 |  | 0.373 |  | -0.022 |  | 0.059 |  |
|  |  | Feeling of Control |  | 0.209 |  | 0.020 |  | 0.299 |  | 10.671 |  | < .001 |  | 0.171 |  | 0.248 |  |
|  |  | Perceived Risk |  | 0.048 |  | 0.025 |  | 0.054 |  | 1.876 |  | 0.061 |  | -0.002 |  | 0.097 |  |
|  |  | Number of Actions |  | 1.943 |  | 0.652 |  | 0.084 |  | 2.980 |  | 0.003 |  | 0.663 |  | 3.222 |  |
|  |  | General Distress |  | -3.424 |  | 0.877 |  | -0.134 |  | -3.904 |  | < .001 |  | -5.145 |  | -1.703 |  |
|  |  | Worry/Fear |  | 0.023 |  | 0.023 |  | 0.033 |  | 1.012 |  | 0.312 |  | -0.021 |  | 0.067 |  |
|  |  | Gender |  | 2.068 |  | 1.140 |  | 0.051 |  | 1.814 |  | 0.070 |  | -0.169 |  | 4.304 |  |
|  |  | Paranoia |  | -5.574 |  | 1.752 |  | -0.104 |  | -3.181 |  | 0.002 |  | -9.013 |  | -2.136 |  |
|  |  | Age |  | 0.032 |  | 0.047 |  | 0.020 |  | 0.678 |  | 0.498 |  | -0.060 |  | 0.123 |  |
|  |  | Stringency index |  | -0.035 |  | 0.036 |  | -0.029 |  | -0.975 |  | 0.330 |  | -0.105 |  | 0.035 |  |
|  | | | | | | | | | | | | | | | | | |

**Logistic regression for “does country fight enough”**

**
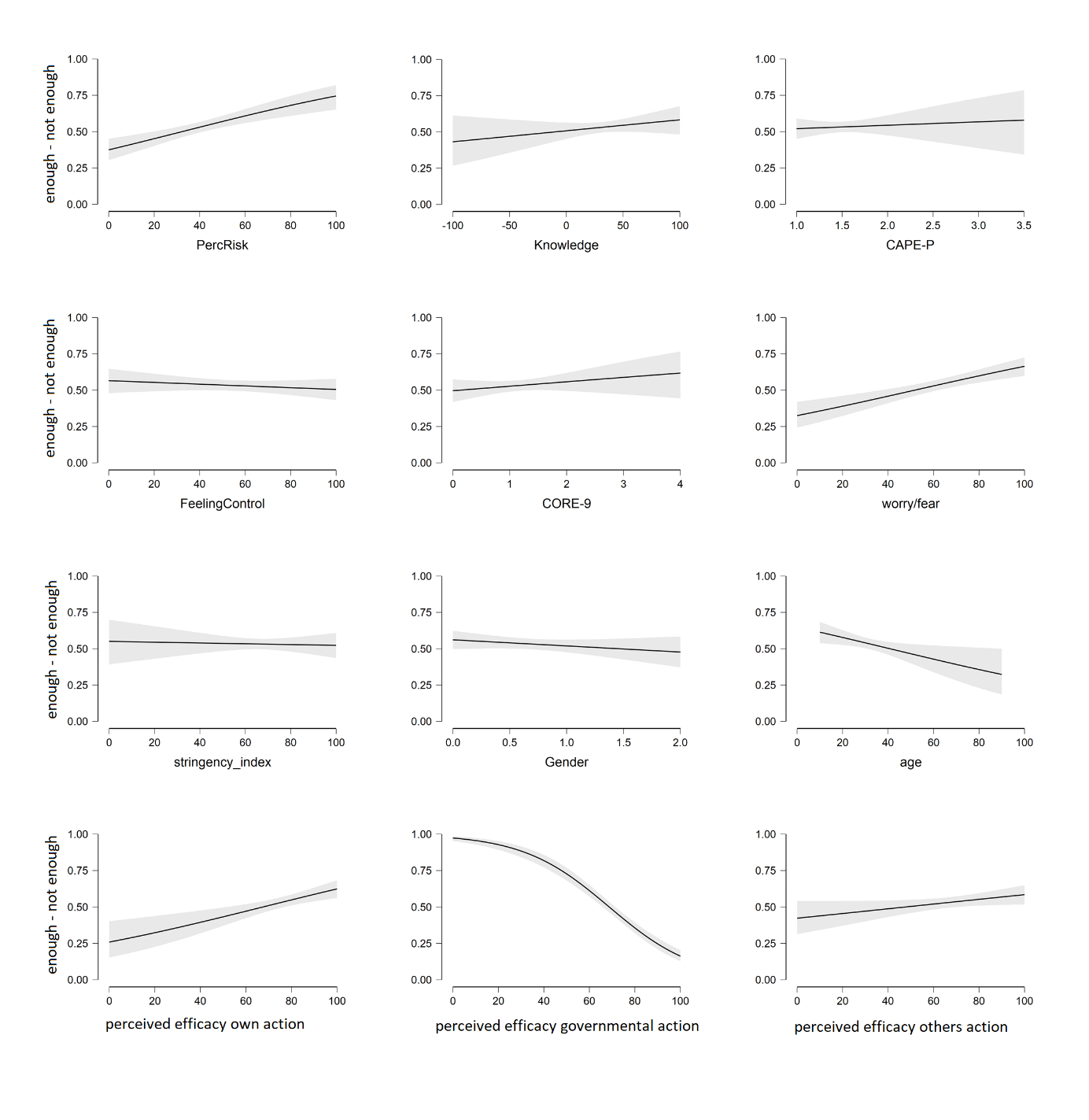
**Figure S3: Conditional estimates plots. If P(not enough) is high, people perceive a higher risk of contracting the virus, are more worried, are younger, perceive their own actions as more effective but that of the government as less effective. CORE-9= General distress, CAPE-P = Paranoia
